# Enhanced germinal center reaction by targeting vaccine antigen to major histocompatibility complex class II molecules

**DOI:** 10.1038/s41541-019-0101-0

**Published:** 2019-02-11

**Authors:** Tor Kristian Andersen, Peter C. Huszthy, Ramakrishna P. Gopalakrishnan, Johanne T. Jacobsen, Marte Fauskanger, Anders A. Tveita, Gunnveig Grødeland, Bjarne Bogen

**Affiliations:** 1 K.G. Jebsen Centre for Influenza Vaccine Research, Institute of Clinical Medicine, University of Oslo, N-0027 Oslo, Norway; 2 Centre for Immune Regulation (CIR), University of Oslo, N-0027 Oslo, Norway; 3 Department of Immunology, Oslo University Hospital, N-0424 Oslo, Norway

## Abstract

Enhancing the germinal center (GC) reaction is a prime objective in vaccine development. Targeting of antigen to MHCII on APCs has previously been shown to increase antibody responses, but the underlying mechanism has been unclear. We have here investigated the GC reaction after targeting antigen to MHCII in (i) a defined model with T and B cells of known specificity using adjuvant-free vaccine proteins, and (ii) an infectious disease model using a DNA vaccine. MHCII-targeting enhanced presentation of peptide: MHCII on APCs, and increased the numbers of GC B cells, T_FH_, and plasma cells. Antibodies appeared earlier and levels were increased. BCR of GC B cells and serum antibodies had increased avidity for antigen. The improved responses required cross-linking of BCR and MHCII in either *cis* or *trans*. The enhanced GC reaction induced by MHCII-targeting of antigen has clear implications for design of more efficient subunit vaccines.

## Vaccine targeting: Enhancement of responses by MHC class II targeting

The germinal center (GC) reaction underpins the robust antibody responses that are the goal of most vaccine approaches. Bjarne Bogen and colleagues at the University of Oslo develop an adjuvant-free engineered vaccine that can enhance the GC reaction. The vaccine is composed of an antibody fragment targeting a mouse major histocompatability complex (MHC) class II (I-E^d^) linked to an experimental myeloma antigen. When tested in B-cell and T-cell receptor transgenic mice specific for the myeloma antigen, GC responses, antibody titers and affinity as well as B and T cell proliferation are all enhanced compared to non-MHC class II targeted vaccine. MHC class II targeting can also enhance antibody responses to influenza antigen using a DNA vaccination approach in wildtype mice. Targeting antigens to MHC class II enhances the GC reaction and has potential implications for the design of more potent and durable vaccines.

## Introduction

Most successful vaccines owe their efficacy to induction of protective antibodies;^1^ indeed, levels of antibodies represent a correlate of protection against most infectious diseases. A key step in development of potent antibodies is the affinity maturation that occurs during the germinal center (GC) reaction. The GC reaction is initiated when activated B cells move into the B-cell follicles.^2^ GC B cells hypermutate the variable (V) region of their B-cell receptor (BCR) through the action of activation-induced cytidine deaminase (AID). Cells with high-affinity BCRs are selected for clonal expansion through interactions with follicular DCs and follicular T-helper cells (T_FH_).^3^ High-affinity B cells develop into long-lived memory B cells and antibody-secreting plasma cells.^4^ For these reasons, increasing GC B-cell responses should be a prime goal in vaccine development.

There is a need to develop efficient platforms for generation of vaccines against rapidly changing and highly pathogenic infectious agents. An attractive approach is the use of subunit vaccines, but these are typically hampered by low immunogenicity. Potentially alleviating this problem, targeting of antigen to cell surface molecules on antigen presenting cells (APCs) can greatly increase T-cell^5–7^ and antibody^8–12^ responses.

Professional APCs include conventional dendritic cells (cDC) type 1 and 2, macrophages, and B cells. These different APC subsets express not only a range of overlapping surface molecules, but also surface molecules that are distinct for each type of APC. In addition, surface expression levels of a given surface molecule may differ greatly between the various APC subsets. These differences are of importance, since the particular surface molecule that is targeted appears to influence the type of immune response that is elicited.^11–14^ For example, targeting of antigen to chemokine receptors 1, 3, and 5 induced predominantly a T-cell response that could mediate broad protection against influenza.^13^ In order to see if the response could even more stringently be polarized to T-cell immunity, we targeted antigen to chemokine XC receptor 1 (Xcr1), exclusively expressed by type 1 cDCs. Such targeting selectively induced Th1/IgG2a responses, in addition to increased CD8^+^ T-cell responses.^5^ We have previously demonstrated that T-cell responses can prevent cancer development,^15^ and confer broad protection against different influenza subtypes.^10,16^ That said, T-cell responses cannot prevent viral entry into host cells, and antibodies remain the most relevant correlate of protection against infectious diseases.

Our side-by-side analysis of nine different APC targets for vaccines has demonstrated that MHCII-targeting was the most efficient for induction of antibodies.^17^ This result is in agreement with our previous studies demonstrating that MHCII-targeted antigen could induce protective antibody responses, both in mice^10^ and larger animals.^18^ In mice, MHCII-targeted hemagglutinin (HA) induced complete antibody-mediated protection within 8 days against challenge with influenza virus, and protective immunity was maintained for at least 10 months.^10^ In order to initiate translation of the promising data with MHCII-targeted vaccination to clinical use, we developed a HLAII-specific targeting unit that could bind HLAII molecules in most humans.^18^ Importantly, the HLAII-specific targeting unit cross reacted with MHCII molecules in larger animals, and we were able to demonstrate that a single DNA vaccination could raise neutralizing antibodies in both ferrets and pigs.^18^ Collectively, these reports demonstrate that MHCII-targeting is associated with particularly rapid and strong antibody responses in a number of different experimental systems across different species. Despite these encouraging results, the immune potentiating mechanism of MHCII-targeting remains unknown.

We have here explored the mechanism by which MHCII-targeting enhances antibody levels, using a highly defined transgenic mouse model, as well as an infectious disease mouse model. The results show that MHCII-targeting augments the GC reaction, resulting in rapid and increased antibody responses of enhanced avidity.

## Results

### Vaccine proteins and model system

**Fig. 1:**
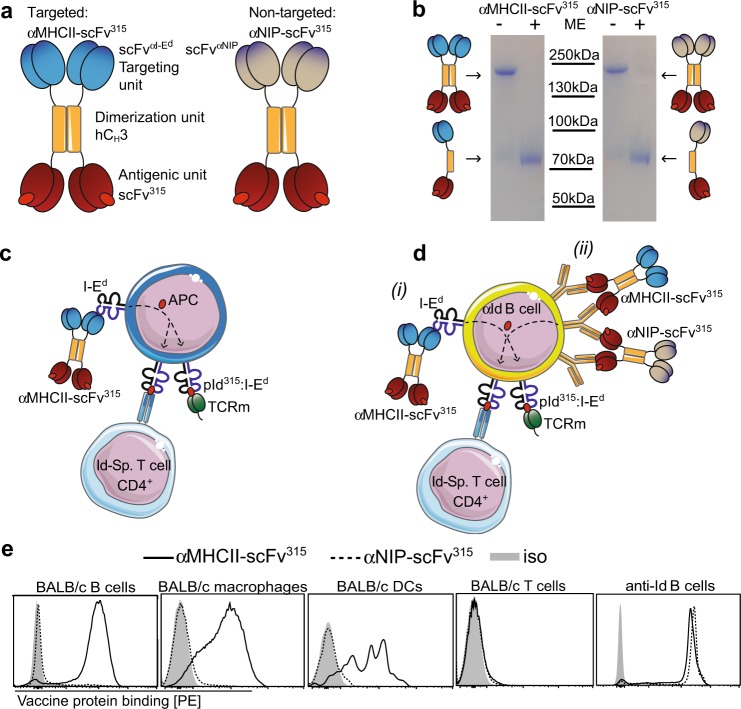
Vaccine proteins and model system. **a** Targeted vaccine proteins have a scFv^αI-Ed^ targeting unit (specific for I-E^d^) linked to the scFv^315^ antigen via a human C_H_3 dimerization domain. In non-targeted vaccine proteins, the scFv^αI-Ed^ has been exchanged with a scFv^αNIP^ specific for the hapten NIP. **b** Purified vaccine proteins analyzed by SDS-PAGE, either with or without β-mercaptoethanol. The displayed bands are from the same gel and were processed in parallel. **c** Targeted vaccine proteins bind to I-E^d^ MHCII molecules on APC, followed by processing and presentation. The scFv^315^ antigenic unit contains an idiotypic(Id)-peptide in the CDR3 loop of its Vλ2 region; this Id-peptide is presented on MHCII molecules (I-E^d^) to Id-specific CD4^+^ T cells from TCR-transgenic mice. In addition, the pId^315^:I-E^d^ complex can be physically recognized by a T-cell receptor mimetic (TCRm) in a scFv format. **d** Model system for testing vaccine proteins in T–B-cell collaboration experiments. B cells from anti-Id BCR knock-in (anti-Id^DKI^) mice have V regions from an anti-Id mAb (Ab2-1.4) that binds to the antigen binding site of scFv^315^. Anti-Id B cells can therefore bind the vaccine protein in two ways: (i) the scFv^αI-Ed^ part of targeted vaccine protein, or (ii) the scFv^315^ antigenic part common to targeted and non-targeted vaccine proteins. Presentation of the Id-peptide on I-E^d^ can be detected either by Id-specific CD4^+^ T cells from TCR-transgenic mice, or by TCRm. **e** Binding of purified vaccine proteins to spleen B cells (CD19^+^), macrophages (F4/80^+^ CD64^+^), DCs (Lin^−^CD11c^hi^), and T cells (CD3^+^) from BALB/c mice, and to anti-Id B cells of anti-Id^DKI^ mice

DNA vaccines encoding fusion proteins that target antigens to MHC class II molecules elicit increased antibody responses.^10,17–19^ To understand the mechanistic basis for these observations, we have here studied vaccine proteins in models with traceable T and B cells of known specificity. Protein delivery was chosen over DNA vaccination since the dose of administered protein could then be directly controlled. The homodimeric vaccine proteins utilized were either targeted against MHCII or an irrelevant hapten (NIP; hereafter referred to as non-targeted vaccine protein), but were otherwise identical. Such an experimental approach allowed us to single out the influence of MHCII-targeting (Fig. 1a, b).

The antigen used was a scFv containing the V_H_ and V_L_ portions of the MOPC315 myeloma protein M315 (termed scFv^315^). When scFv^315^ is taken up by APCs, an idiotypic (Id) peptide derived from the CDR3 loop of Vλ2^315^ is generated by intracellular processing, and is presented by cell surface MHCII (I-E^d^).^20^ The pId^315^:I-E^d^ complex is recognized by Id-specific CD4^+^ T cells from TCR-transgenic mice,^21^ but can also be physically detected by a TCR mimetic (TCRm), a scFv generated by phage display technology recently developed in our lab (Fig. 1c).

Experiments were performed using different types of APCs (DC, MΦ, B cells). The MHCII-specific vaccine proteins should interact with these different APCs by binding to their cell surface MHCII (Fig. 1c). The exception is when using scFv^315^-specific (anti-Id) B cells from anti-Id BCR knock-in (anti-Id^DKI^) mice.^22^ The latter cells should be able to bind to the vaccine proteins in two different ways: (i) via the scFv^αI-Ed^ moiety of the MHCII-targeted vaccine protein, or (ii) via the scFv^315^ antigen shared by the targeted and non-targeted vaccine versions (Fig. 1d). In addition, a single MHCII-targeted vaccine protein could simultaneously bind the BCR and I-E^d^ on a single anti-Id B cell. These above deliberations were confirmed by staining experiments, which demonstrated that the MHCII-targeted vaccine proteins bound to B cells, macrophages, and DCs (but not T cells) from BALB/c mice, while the non-targeted control vaccine protein failed to bind any of these cell types (Fig. 1e). By contrast, either of the two vaccine proteins bound anti-Id B cells (Fig. 1e). Binding of vaccine proteins to MHCII molecules was also confirmed with L-cell transfectants (Supplementary Fig. 1).

### MHCII-targeting enhances signaling and peptide: MHCII presentation

**Fig. 2:**
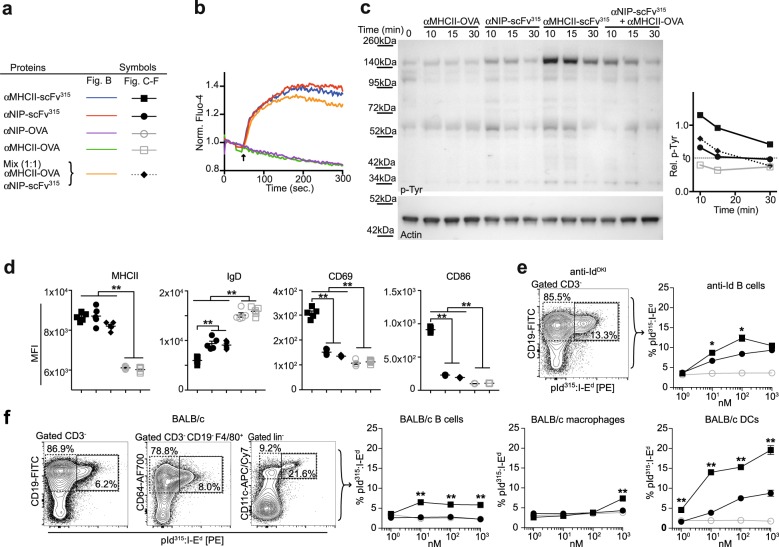
MHCII-targeting enhances signaling and peptide:MHCII presentation. **a** Symbols. **b**, **c** Response of negatively enriched anti-Id B cells in the presence of 500 nM of the indicated vaccine proteins. **b** Calcium flux. The arrow indicates time point of added ligand. **c** Phosphotyrosine levels at indicated time points measured in western blot. Relative phosphotyrosine levels quantified from the blot are shown as the area under the peaks normalized to loading control. The blots are from the same experiment and were processed in parallel. **d** Anti-Id B cells were incubated with 1 nM of vaccine proteins for 20 h and analyzed for expression of the indicated surface markers in flow cytometry. **e**, **f** Splenocytes from anti-Id^DKI^ or BALB/c mice were incubated for 16 h with titrated doses of the indicated vaccine proteins. Surface expression of pId^315^:I-E^d^ complex was detected with a TCRm on the indicated cell types. **e** Detection of pId^315^:I-E^d^ on anti-Id B cells. **f** Detection of pId^315^:I-E^d^ on BALB/c B cells, macrophages, and DCs. Flow panels in **e**, **f** show pId^315^:I-E^d^ signal after incubation with 1 µM targeted protein and gating for cell subset (dashed) and pId^315^:I-E^d^ positive signal (solid). All experiments are representative of two or three independent experiments. **d**–**f** *n* = 5 per group. Mean ± SEM. *p < 0.05 and **p < 0.01, (**e**–**f**; αMHCII-scFv^315^ vs. αNIP-scFv^315^) unpaired two-tailed Student’s *t* test

Initial responses of antigen-specific B cells to vaccine proteins were evaluated by measuring calcium flux and phosphorylation at early time points in negatively selected anti-Id^DKI^ B cells (Supplementary Fig. 2a). Vaccine proteins with irrelevant antigen (OVA) were used as controls. The results show that ligation of BCR alone induced both a calcium flux and protein phosphorylation, as would be expected, while ligation of MHCII alone did not induce any responses (Fig. 2b, c). Vaccine proteins that could simultaneously ligate both BCR and MHCII resulted within 10 min in a striking increase in phosphorylation over that obtained with BCR ligation alone (Fig. 2c and Supplementary Fig. 3). This effect required a linkage between the BCR ligand and the MHCII-specific moiety, since an equimolar mix of MHCII-targeted OVA and non-targeted scFv^315^ failed to increase phosphorylation.

Next, we tested if the different vaccine proteins could enhance activation of anti-Id B cells during a longer incubation period. After 20 h of incubation in the presence of non-targeted vaccine scFv^315^ protein, anti-Id B cells upregulated MHCII and downregulated IgD. In the presence of MHCII-targeted scFv^315^, a further decrease in IgD expression was observed. In addition, MHCII-targeting strikingly increased CD69 and CD86 (Fig. 2d). As observed for phosphorylation above, separate ligation of MHCII and BCR did not synergize, demonstrating that physical linkage of targeting- and antigenic moiety is required to augment B-cell activation.

In order to measure the effect of targeting on MHCII peptide presentation on APCs, we utilized a TCRm that specifically recognizes the pId^315^:I-E^d^ complex. Splenocytes from anti-Id^DKI^ mice or BALB/c mice were incubated with titrated amounts of vaccine proteins, followed by flow cytometric measurement of pId^315^:I-E^d^ complexes on B cells, macrophages, and DCs. For anti-Id B cells, incubation with MHCII-targeted vaccine proteins resulted in a significantly higher display of pId^315^:I-E^d^ complexes as compared with incubation with non-targeted vaccine proteins (Fig. 2e). When tested with BALB/c B cells, only the MHCII-targeted vaccine increased the display of pId^315^:I-E^d^ complexes, while non-targeted vaccine protein had no effect (Fig. 2f). However, the expression level of pId^315^:I-E^d^ complexes on BALB/c B cells was reduced to ∼50% of that observed for anti-Id B cells. Thus, binding of the vaccine protein to both BCR and MHCII (Fig. 1d) appeared to synergistically contribute to the display of pId^315^:I-E^d^ complexes. BALB/c DCs incubated with vaccine proteins exhibited the highest display of pId^315^:I-E^d^ complexes; the targeted version being about 1–2 log more efficient than the non-targeted control, as evaluated from the dose–response curves (Fig. 2f). Macrophages stained poorly with the TCRm, and expression was only detectable after exposure to the targeted vaccine protein (Fig. 2f). In summary, MHCII-targeting of antigen increased signaling, activation, and display of p:MHCII on antigen-specific B cells.

### Targeting antigen to MHC class II molecules increases proliferation of T and B cells in vitro

**Fig. 3:**
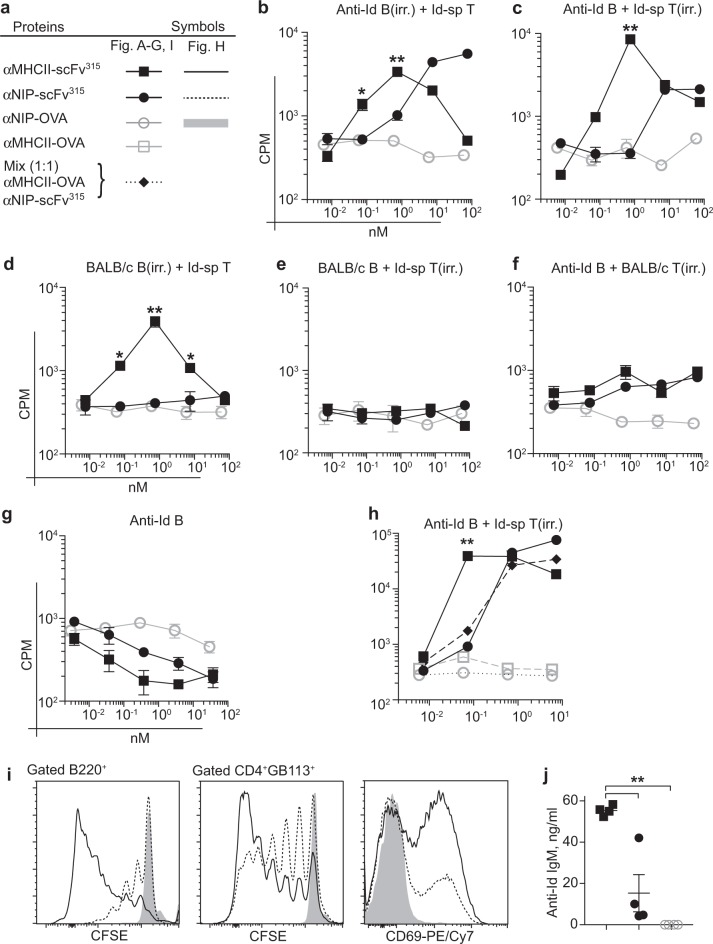
Targeting antigen to MHC class II molecules increases proliferation of T and B cells in vitro. **a** Symbols. Naive T and B cells were enriched by negative selection from the spleens of TCR Tg and anti-Id^DKI^ mice (Supplementary Fig. 2), or BALB/c mice. **b**–**h** Either T cells or B cells were irradiated (irr.), and indicated mixtures of 5 × 10^4^ T cells and 1 × 10^5^ B cells were seeded with titrated amounts of indicated vaccine proteins. Proliferation was assayed by ^3^HTdR incorporation. **i**, **j** Id-specific T cells and anti-Id B cells were CFSE-labeled and cultured (1:1, 5 × 10^5^) together with 1 nM of the indicated vaccine proteins for 5 days. **i** Flow cytometry analysis of CFSE signal and expression of CD69 on Id-specific T cells. **j** Anti-Id IgM levels in supernatant. All experiments are representatives from single experiments repeated two or three times. **b**–**h** *n* = 3 per group. **j** *n* = 4 per group. Mean ± SEM. *p < 0.05 and **p < 0.01, (**b–h**; αMHCII-scFv^315^ vs. αNIP-scFv^315^) unpaired two-tailed Student’s *t* test

Naive, Id-specific T and B cells have previously been shown to collaborate efficiently in the presence of Id^+^ Ig, even in the absence of DCs.^22^ Here, we enriched B cells (BALB/c or anti-Id) and T cells (BALB/c or Id-specific from TCR-transgenic mice; Supplementary Fig. 2b), and mixtures of cells were assayed for proliferative responses to the MHCII-targeted and non-targeted versions of the vaccine proteins. Either T cells or B cells were irradiated in order to quantify proliferative responses of the counterpart. Antigenic potencies of vaccine proteins were estimated from the descending slopes of dose–response curves at diminishing concentrations (at higher concentrations, inhibition was observed, as commonly seen in these types of assays). In co-cultures containing both Id-specific T cells (Fig. 3b) and anti-Id B cells (Fig. 3c), both cell types responded to MHCII-targeted and non-targeted proteins. However, responses against the targeted version were significantly stronger (∼×10) than those against the non-targeted version. In mixtures of BALB/c B cells and Id-specific T cells, only MHCII-targeted protein induced proliferation (Fig. 3d), consistent with the TCRm staining in Fig. 2f. Further, since only T cells and not B cells responded to MHCII-targeted protein, B cells appear to require BCR ligation in addition to T cell help for proliferation (Fig. 3e–g).

Given the finding that MHCII-targeting increased antigen-specific T- and B-cell responses, we proceeded to test whether physical linkage between the αMHCII and scFv^315^ moieties of the vaccine protein was required for the augmenting effect (Fig. 3h). Since a mixture of αMHCII-OVA and αNIP-scFv^315^ proteins did not increase responses above that of αNIP-scFv^315^ alone, it can be concluded that the αMHCII and scFv^315^ moieties need to be linked in order to enhance B- and T-cell responses.

To exclude any possible effects of irradiation, we repeated the experiments with CFSE-labeled anti-Id B cells and Id-specific T cells (Fig. 3i). MHCII-targeted vaccine proteins elicited more vigorous proliferation in both B cells and T cells, as well as an upregulation of the T-cell activation marker CD69, when compared with the non-targeted version. The MHCII-targeted vaccine proteins apparently elicited some differentiation into antibody-secreting cells, since MHCII-targeting increased IgM levels in supernatants over that of non-targeted controls (Fig. 3j). In summary, MHCII-targeting of the antigen clearly increased antigen-specific T- and B-cell responses in vitro, and physical linkage of the targeting moiety and antigen was required.

### MHCII-targeting of antigen increases antibody responses in vivo

**Fig. 4:**
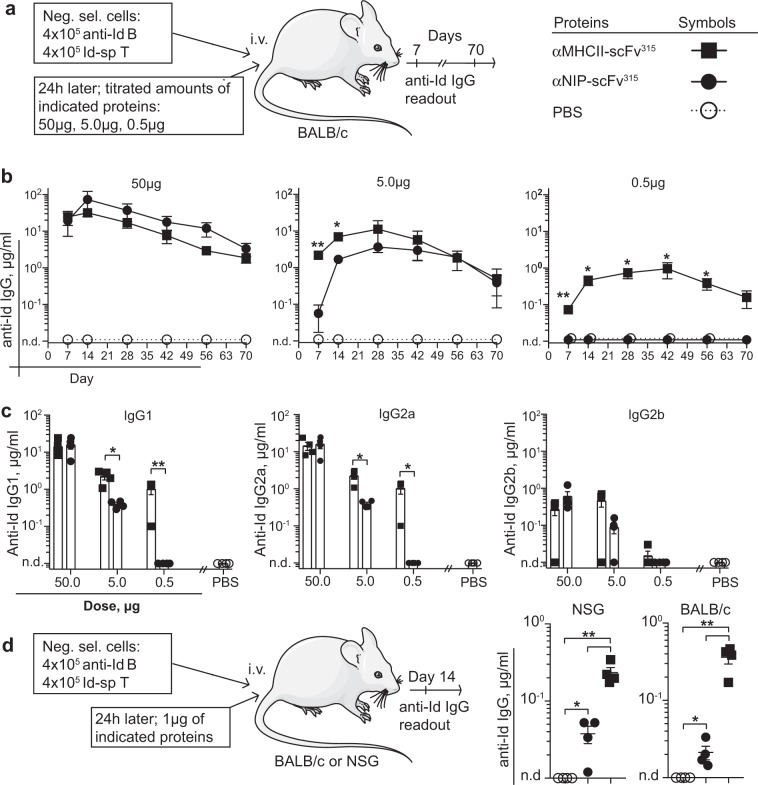
MHCII-targeting of antigen increases antibody responses in vivo independent of dendritic cells. **a** Experimental layout and symbols. **b** Anti-Id IgG levels in sera over time in groups receiving different amounts of vaccine proteins. **c** Anti-Id IgG1, IgG2a, and IgG2b levels in serum on day 14. **d** Experimental layout using either NSG or BALB/c mice as recipients. Anti-Id IgG levels in sera were measured on day 14. *n* = 4 mice per group. Mean ± SEM. *p < 0.05 and **p < 0.01, (b; αMHCII-scFv^315^ vs. αNIP-scFv^315^) unpaired two-tailed Student’s *t* test

To determine the in vivo relevance of targeted antigen delivery, Id-specific T cells and anti-Id B cells were transferred i.v. to unirradiated BALB/c recipients, followed by titrated amounts of vaccine proteins injected i.v. 24 h later (Fig. 4a). Anti-Id antibodies in the sera were quantified over the ensuing 10 weeks. The results demonstrate that the potentiating effect of MHCII-targeting was more significant at lower doses of vaccine protein (Fig. 4b). More specifically, while 5.0 µg and 0.5 µg of MHCII-targeted vaccine proteins significantly increased antibody responses compared with the non-targeted version, no difference was seen with the 50 µg dose. For the 5.0 µg dose, MHCII-targeting significantly increased anti-Id IgG levels only during the first 2 weeks, as compared with the non-targeted control vaccine. Furthermore, for the 0.5 µg dose, only MHCII-targeted protein induced anti-Id IgG levels, while the non-targeted control failed to elicit any response at all. For the 0.05 μg protein dose, neither vaccines induced detectable antibody responses (Supplementary Fig. 4a).

Various anti-Id IgG isotypes (IgG1, IgG2a, IgG2b, and IgG3) were measured on day 14 post-vaccine administration (Fig. 4c). In line with the above data, the difference between MHCII-targeted and non-targeted antigens became more significant with decreasing amounts of vaccine proteins injected. The anti-Id antibody response was dominated by IgG1 and IgG2a. More limited amounts of IgG2b were observed, while no IgG3 could be detected (Supplementary Fig. 4b).

### Antibody responses elicited by MHCII-targeting do not require presentation by dendritic cells

Since DC efficiently presented pId^315^:I-E^d^ complexes after MHCII-targeting in vitro (Fig. 2), we wanted to investigate the contribution from such primed DCs in the enhanced T–B collaboration elicited by MHCII-targeting in vivo. To this end, we took advantage of immunocompromised NOD-SCID common γ_c_ knockout (NSG) mice, which have an H-2^g7^ MHC haplotype. NSG mice only express a single MHC class II molecule of the I-A isotype, I-A^g7^, and do not express I-E molecules. Thus, the scFv^αI-Ed^ targeting unit, derived from the Eα-specific 14-4-4S mAb, should not bind DC in NSG mice. This was verified by flow cytometry (Supplementary Fig. 5). Since NSG mice are severely immunocompromised, they may be readily engrafted with cells from other mouse strains. Purified Id-specific T cells and anti-Id B cells were transferred to NSG and BALB/c mice, followed by MHCII-targeted or non-targeted vaccine proteins 24 h later. The presence of anti-Id Ig was assessed in sera 2 weeks later. The levels of anti-Id IgG obtained were indistinguishable in NSG and BALB/c mice (Fig. 4d), and the MHCII-targeting effect (as seen previously) was pronounced in both cases. These results indicate that DCs are not required for the antibody responses observed with MHCII-targeting.

### Increased proliferation of T and B cells in vivo after MHCII-targeting of antigen

**Fig. 5:**
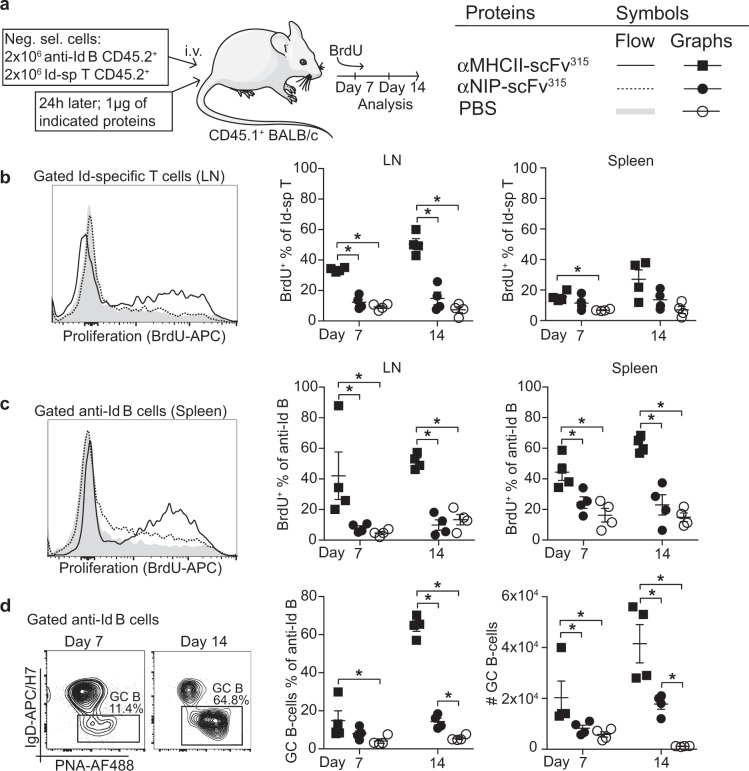
Increased proliferation of T and B cells in vivo after MHCII-targeting of antigen. **a** Experimental layout and symbols. Transfer of congenically marked T and B cells (Supplementary Fig. 6b). **b** Proliferation of Id-specific T cells in LNs and spleen. **c** Proliferation of anti-Id B cells in LNs and spleen. **d** Fractions and absolute numbers of anti-Id B cells with a GC phenotype (PNA^hi^ IgD^lo^) in the spleen 7 and 14 days after immunization. *n* = 4 per group. Mean ± SEM. *p < 0.05; two-tailed Mann–Whitney test

The enhanced IgG response seen after injection of MHCII-targeted proteins suggests an expansion of antigen-specific T and B cells. To test this, Id-specific T cells and anti-Id B cells were transferred i.v. to CD45.1 congenic BALB/c mice (Supplementary Fig. 6b), followed by delivery of either MHCII-targeted or non-targeted vaccine proteins, and continuous exposure to BrdU. The spleen and lymph node (LN) suspensions were analyzed 7 and 14 days later. Transferred T and B cells were identified as CD45.2^+^CD4^+^ and CD45.2^+^B220^+^ lymphocytes, respectively (Fig. 5a). Analysis of BrdU incorporation in Id-specific T cells demonstrated a significantly increased proliferation elicited by MHCII-targeted proteins (Fig. 5b). Proliferation could be observed already on day 7 in LN, with a further increase by day 14. Markedly less T-cell proliferation was found in the spleen (Fig. 5b). Similarly, MHCII-targeting of antigen significantly increased the numbers of proliferating B cells both at days 7 and 14 after vaccination, in the LNs as well as in the spleen (Fig. 5c). The difference in T- and B-cell proliferation in LNs and the spleen likely reflects lymphocyte residence in the host with LNs being rich in T cells. Further, MHCII-targeting resulted in an increased fraction (and absolute numbers) of anti-Id B cells that showed a GC phenotype (PNA^high^ IgD^low^) on day 7 with a further enhancement by day 14. In contrast, the non-targeted control vaccine protein did not increase the levels of GC B cells above the PBS control until day 14, at which time point a significant difference was detected (Fig. 5d).

### MHCII-targeting of antigen increases the GC response

**Fig. 6:**
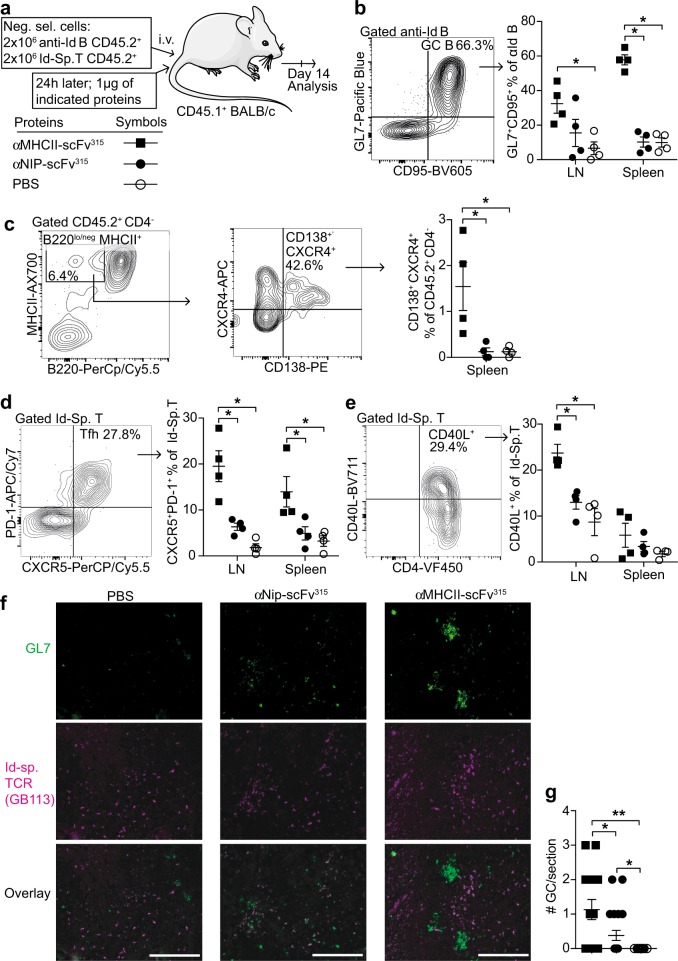
MHCII-targeting of antigen increases the GC response. **a** Experimental layout and symbols. **b** Gating strategy for GC B cells (left panel) and their quantification (right). **c** Identification of early plasma cells (CD45.2^+^ CD4^−^B220^lo^ MHCII^hi^ CXCR4^+^ CD138^+^) and their quantification. **d** Gating of T_FH_ cells and their quantification. **e** Upregulation of CD40L on Id-specific T cells. **f** Representative micrographs of immunostained cryosections of spleens. Scale bar is 200 µm. **g** Quantification of GCs with GL7^+^ cells and interspersed Id-specific T cells. 120 10x fields were counted in total per group and represented as number of GC per cryosection of spleen. *n* = 4 per group. Mean ± SEM. *p < 0.05 and **p < 0.01, two-tailed Mann–Whitney test

Next, we tested in more detail if MHCII-targeting could increase the GC reaction. Naive Id-specific T and anti-Id B cells were transferred into CD45.1 congenic BALB/c mice, and vaccine proteins were injected 24 h later (Fig. 6a). Fourteen days later, spleens and LN suspensions were analyzed. GC B cells were identified as B220^+^ GL7^+^CD95^+^. The proportion of anti-Id B cells with a GC phenotype increased upon MHCII-targeting, as compared with the non-targeted control (Fig. 6b). The GC B-cell responses were stronger in the spleen than in LN. In the spleen, a significant increase in early plasma cells was detected in mice receiving MHCII-targeted antigen (Fig. 6c). As for T_FH_, the MHCII-targeted protein significantly enhanced their abundance both in the spleen and in the LNs (Fig. 6d). Moreover, MHCII-targeting significantly increased the expression of CD40 ligand on Id-specific T cells, as compared with the non-targeted control (Fig. 6e).

Sections of spleens from the above experiment were stained with the GC marker GL7 and the anti-clonotypic mAb GB113 (reactive against the TCR of Id-specific T cells) (Fig. 6f). GCs with intrafollicular accumulations of GL7^+^ cells and interspersed GB113^+^ cells were increased in sections from mice that had received MHCII-targeted antigen, compared with mice that had received either the non-targeted control or PBS (Fig. 6g). Thus, both flow cytometry and immunohistochemistry analysis supported the notion that MHCII-targeting increases the GC reaction.

### MHCII-targeting of influenza hemagglutinin delivered by a DNA vaccine increases germinal center differentiation of B cells

**Fig. 7:**
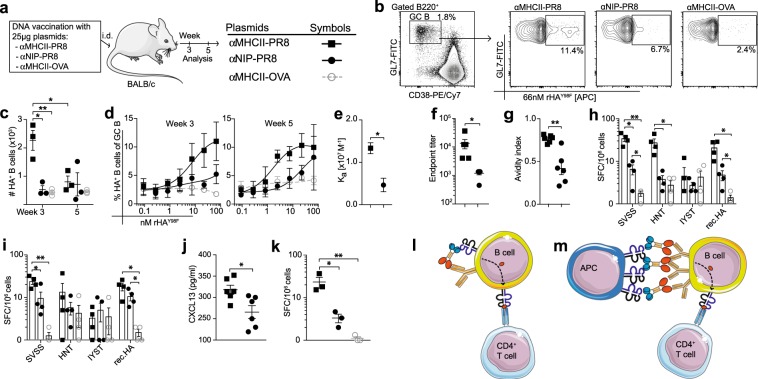
MHCII-targeting of influenza hemagglutinin delivered by a DNA vaccine increases germinal center differentiation of B cells. **a** Experimental layout and symbols. BALB/c mice were vaccinated i.d. with the indicated plasmid DNA constructs followed by electroporation. Draining LNs were harvested at 3 and 5 weeks post vaccination. **b** Gated GC B cells were stained with recombinant rHA^Y98F^ (66 nM) (Supplementary Fig. 7). Representative data are shown. **c** Absolute numbers of HA-reactive GC B cells in draining LNs. **d** Gated GC B cells from week 3 and 5 were stained with serially diluted rHA^Y98F^ probe. **e** BCR avidity measurements at week 5 with affinity constant determined from non-linear curve fitting of binding curves in **d**. **f** Endpoint titers of HA-specific IgG in serum at week 5. **g** Avidity of week 5 serum anti-HA IgG determined by Urea-wash ELISA. **h**–**i** Lymphocytes collected at week 3 were stimulated with MHCII-(SVSSFERFEIFPK, HNTNGVTAACSHEG) or MHCI- (IYSTVASSL) restricted HA peptides or rHA, and the number of IL-4 **h** and IL-21 **i** secreting cells determined by ELISPOT. **j** Sera collected at week 3 were assayed for CXCL13 by ELISA. **k** Quantification of bone marrow HA-specific plasma cells at week 5. **l**–**m** Schematic representation of two possible mechanisms for enhanced B-cell responses observed by MHCII-targeting. **l** Cross-linking of BCR and MHC class II on a single B cell. **m** Vaccine proteins could form a bridge between an APC and a B cell, in an APC-B-cell synapse. **c–e**, **k** *n* = 3 per group, **f**–**g**, **j** *n* = 6 per group, **h**–**i** *n* = 4 per group. Mean ± SEM. *p < 0.05 and **p < 0.01 **e** extra sum-of-squares F test, **c**, **f–k** unpaired two-tailed student’s *t* test

The above experiments were done in a highly defined experimental model with a tumor antigen, myeloma protein V regions. We wished to generalize the observations to ordinary inbred strains of mice with a relevant infectious disease antigen. Moreover, we sought to extend the observations to DNA vaccination, which might represent a more realistic vaccine approach for mass vaccinations.^23^ With these goals in mind, BALB/c mice were immunized with plasmid DNA constructs encoding homodimeric proteins that targeted either HA^10^ or OVA^13^ to MHC class II molecules on APCs. A non-targeted control specific for the hapten NIP was included (Fig. 7a). In order to detect HA-specific B cells, a recombinant HA (rHA^Y98F^) probe was developed. In this probe, to minimize non-specific binding, tyrosine was substituted with phenylalanine at position 98, which abolishes binding to sialic acid (SA), the cellular receptor of HA (Supplementary Fig. 7).^24^ Staining of GC B cells (B220^+^GL7^+^CD38^−^) on weeks 3 and 5 following vaccination demonstrated that the rHA^Y98F^ probe could efficiently stain HA-reactive B cells^25^ (Fig. 7b). Significantly higher numbers of HA-reactive GC B cells were detected at week 3 after MHCII-targeted immunization, compared with the non-targeted control. The number of HA-specific GC B cells was higher on week 3 than on week 5, probably due to an involution of the anti-HA response (Fig. 7c). For an assessment of the avidity of HA-specific B cells, lymphocytes were stained with serially diluted rHA^Y98F^ protein (Fig. 7d). Since high-affinity B-cell clones can bind antigens at lower concentrations, the titration curves indicate the avidity of HA-specific GC B cells.^25^ The results show that avidity of the HA-specific GC B cells was enhanced by MHCII-targeting (Fig. 7e). Consistent with the increased number and avidity of the GC B cells, MHCII-targeting increased titers and antigen avidity of serum antibodies at week 5 (Fig. 7f–g). In summary, MHCII-targeting of HA significantly increased both the numbers and the avidity of GC B cells, as compared with the control vaccines (αNIP-HA and αMHCII-OVA).

As an indicator of T cell help to B cells,^26^ we investigated the number of IL-4 and IL-21 secreting cells in the LNs at week 3. Lymphocytes stimulated with either MHCII-restricted peptides or full-length recombinant HA showed a significant elevation of IL-4 (Fig. 7h) and IL-21 (Fig. 7i) secreting cells after MHCII-targeted immunization, in contrast to vaccination with controls (αNIP-HA or αMHCII-OVA). Furthermore, the significantly higher serum levels of CXCL13 seen in mice vaccinated with αMHCII-HA, as compared with the non-targeted control, offered further evidence that targeting of antigen to MHCII molecules enhanced the GC reaction^27^ (Fig. 7j). Finally, quantification of antigen-specific plasma cells in the bone marrow at week 5 post immunization demonstrated that MHCII-targeted vaccination significantly increased the number of anti-HA antibody-secreting plasma cells, as would be expected from an increased GC reaction^4^ (Fig. 7k).

## Discussion

Enhancing the GC reaction is a prime objective in vaccine development against infectious diseases. Here, we show that targeting of antigen to MHCII results in an enhanced GC reaction, manifested by increased numbers of antigen-specific GC B cells that display a higher avidity. Follicular T helper cells, plasma cells, and antibody levels were also augmented. The experiments were performed in a highly defined model with BCR knock-in B cells and TCR-transgenic T cells,^21,22^ which enabled a high resolution of cellular and molecular mechanisms. Supporting observations were made in wild-type mice immunized with an MHCII-targeted influenza hemagglutinin DNA vaccine, suggesting that the findings may be of general relevance.

An important tool in these studies was a TCRm that allows direct detection of an antigenic peptide bound to MHC class II molecules (p:MHCII). Staining of cells from BALB/c mice with TCRm revealed that MHCII-targeted vaccine proteins resulted in increased display of p:MHCII, especially on DCs, but also on B cells, which translated into enhanced T-cell responses. These observations imply that MHC class II molecules can serve as endocytic receptors, independent of BCR, resulting in enhanced generation of p:MHCII for display to CD4^+^ T cells.

When antigen-specific BCR knock-in B cells were used, both the MHCII-targeted and non-targeted vaccine proteins resulted in display of p:MHCII, as would be expected from the known endocytic function of BCRs. However, MHCII-targeting significantly enhanced the p:MHCII display, resulting in increased T-cell stimulation compared with the non-targeted control. For such an enhancement to occur, the MHCII-binding moiety and the antigen had to be linked in a single molecule, indicating that cross-linking of BCRs and MHC class II molecules is essential for the increased efficiency of the targeted vaccine proteins. The cross-linking may occur in *trans* (between two cells) or in *cis* (intrinsic to a B cell), as discussed below. The two possibilities are not mutually exclusive.

The potentiating effect of cross-linking BCR and MHCII could stem from the formation of APC-B-cell synapses (*trans*), as previously suggested.^28^ Consistent with this idea, antibody responses are triggered when B cells bind antigen on APCs, such as dendritic cells^29,30^ or subcapsular sinus macrophages.^31,32^ Moreover, soluble antigens are several 100-fold more immunogenic when forming immune complexes that are associated with membranes via complement- or Fc-receptors.^33–35^ Furthermore, there is evidence to suggest that B cells acquire antigen in APC-B-cell synapses.^36,37^ Finally, B-cell interactions with membrane-bound antigen on follicular DCs in the draining LNs are essential during the affinity maturation process.^38–40^ Based on these previous results, it is reasonable to speculate that immuniziation with MHCII-targeted vaccine protein enhances B-cell interaction with the APC-displayed antigen, thereby increasing B-cell responses.

In an APC-B-cell synapse (*trans*), any of the many types of MHCII^+^ cells (e.g., cDC1, cDC2, macrophages, B cells) could in principle serve as APC. Relevant to this, transfer of Ag-specific T and B cells into immunodeficient NSG recipients, whose MHCII molecules are not bound by the targeting moiety of the vaccine proteins used herein, revealed that the increased GC reaction elicited by MHCII-targeting could occur in the absence of other APCs than B cells. This finding does indeed not exclude an unspecific (e.g., growth-promoting) role of non-B-cell APCs. Two non-exclusive possibilities exist for responses in the absence of targeted DC: MHCII-targeted Ag could (i) cross-link BCR and MHC class II on single B cells in *cis* (Fig. 7l), or (ii) cross-link two different B cells in *trans*, binding MHCII on one B cell and BCR on the other, similar to a conventional APC-B-cell synapse (Fig. 7m).

The initial recruitment of B cells to GCs is broad, and early GCs contain B cells with a wide range of affinities.^41–43^ It is possible that targeting of antigen to MHCII could increase the initial recruitment of B cells into GCs, especially if cross-linking of MCHII and BCR can activate B cells that would not otherwise be activated by antigen alone.^44^ Thus, MHCII-targeted antigen can initiate immune responses at doses where non-targeted antigen is unreactive. Later in the GC reaction, high-affinity B cells are selected and amplified by affinity-driven competition between B cells that have acquired somatic mutations in their V regions.^43,45^ A selective advantage of high-affinity B cells may be conferred by increased BCR-mediated accumulation of antigen, increased display of p:MHCII on the B cell’s surface, and increased B-cell reception of T-cell help.^46,47^ MHCII-targeted immunization at medium doses might accelerate induction of antibodies due to increased presentation of p:MHCII intrinsic to MHCII-targeting, while delivery of non-targeted antigen more strictly depends on BCR affinity maturation to capture and present p:MHCII on B cells. The present results appear to support this idea since MHCII-targeting enhances p:MHCII display on GC B cells and augments T_FH_ numbers at low doses.

We have previously shown that a single DNA vaccination of mice with MHCII-targeted HA confers antibody-mediated sterilizing immunity against a lethal challenge with influenza virus, while non-targeted controls fail to do so.^10^ Further, MHCII-targeted DNA vaccines improve antibody responses in ferrets and pigs.^18^ Here, we show that MHCII-targeting increases the GC reaction as indicated by elevated numbers of HA-reactive GC B cells, enhanced CXCL13 levels in plasma,^27^ and augmented levels of IL-4 and IL-21 secreting cells.^26^ Moreover, and importantly, ΜΗCII-targeted DNA immunization resulted in GC B cells and serum antibodies with a significantly higher avidity for HA. These results indicate that DNA formats which target protein antigens to MHCII might overcome problems with weak immunogenicity of DNA vaccines. The MHCII-targeted approach may become important since DNA vaccines, due to their rapid construction and production, are attractive in epidemic and pandemic settings.

Enhancing the GC reaction, similar to that obtained herein with MHCII-targeting of antigen, is a clear goal in development of novel vaccines for a number of infectious diseases. This might be particularly so for influenza and HIV vaccines intended to induce broadly neutralizing antibodies, since such antibodies express exceptionally high loads of mutations in their V regions.^48,49^

## Methods

### DNA and proteins

pLNOH2 plasmid vectors encoding the different vaccines were used for transfections and in vivo immunizations. The encoded vaccine proteins contained either a single-chain variable fragment (scFv) specific for MHCII (I-E^d^) (targeting unit), or for the hapten 4-hydroxy-3-iodo-5-nitrophenylacetic acid (NIP) (non-targeted control); and the antigenic units consisted of either (1) a scFv containing the idiotope of λ2^315^ found in the CDR3 fragment of the λ2^315^ light chain in Id^+^ M315 IgA (scFv^315^), (2) ovalbumin (OVA), or (3) hemagglutinin (HA) from the A/Puerto Rico/8/1934 (H1N1) influenza strain. All protein vaccines were produced by transient transfection of HEK 293E cells using polyethylenimine-complexed DNA, followed by affinity purifications. The scFv^315^ constructs were purified using a DNP-coated affinity chromatography column produced in the laboratory. Ovalbumin constructs were purified using a CaptureSelect FcXL affinity chromatography column (194328005, Life Technologies, Naarden, The Netherlands). DNA preparations used for in vivo immunizations were endotoxin-free (purified with EndoFree Plasmid Mega Kit 12381, Qiagen, Hilden, Germany).

### Enrichment of T and B cells

Cells were enriched by negative selection from spleens of anti-Id^DKI^ mice, TCR-Tg mice, or BALB/c mice using anti-mouse CD43 (Untouched™ B Cells) kit (11422D, Invitrogen, Oslo, Norway) or Untouched™ Mouse CD4 Cells Kit (11415D, Invitrogen).

### In vitro proliferation assay

Enriched T and B cells (5 × 10^4^ T cells and 1 × 10^5^ B cells in 96-well plates for radiolabel assays, and 1 × 10^5^ T and 1 × 10^5^ B cells in 96-well plates for CFSE-label assays) were seeded together in triplicates in RPMI-1640 medium (R2405-500ML, Sigma-Aldrich, St. Louis, MO, USA) with 10% fetal calf serum (heat inactivated) with added stimulating proteins or controls. In radiolabel assays, either the T- or the B-cell counterpart was inactivated by irradiation (15 Gy, XSTRAHL LTD, Camberley, UK). In total, 25 µCi [^3^H]TdR (MT6032, Hartmann Analytic, Braunschweig, Germany) was added the last 16 h of a 64 h culture before reading with a MicroBeta plate reader (PerkinElmer, Waltham, MA, USA). CFSE label assays were incubated for 5 days and analyzed using an Attune NxT flow cytometer (Thermo Fisher Scientific, Waltham, MA, USA).

### Mice

Six-to-eight weeks old female BALB/c (Janvier, le Genest-Saint-Isle, France), CByJ.SJL(B6)-Ptprca/J (Jackson Laboratory, Bar Harbour, ME, USA), or NOD.Cg-*Prkdc*^*scid*^ *Il2rg*^*tm1Wjl*^/SzJ (Jackson Laboratory) were used. Anti-Id^DKI^ mice have B cells with V regions from an anti-Id mAb (Ab2-1.4), i.e., anti-Id BCR knock-in (anti-Id^DKI^) mice on BALB/c background.^22^ Id-specific TCR-Tg mice are αβ TCR-transgenic BALB/c with T cells recognizing λ2^315^ antigenic peptide on I-E^d^, i.e., pId^315^:I-E^d^- specific TCR^21^. The animals were housed in the Minimal Disease Unit in our institute. The in vivo procedures were approved by the Norwegian Animal Research Authority.

### In vivo cell and protein transfer, BrdU feeding, and DNA vaccination

Negatively selected, presumably naive, T and B cells were injected intravenously to naive BALB/c or CByJ.SJL(B6)-Ptprca/J mice. Twenty-four hours after cell transfer, the mice received vaccine proteins or PBS intravenously. Mice received 1 mg BrdU by intraperitoneal injection the day after protein transfer, and also 5–7 days later depending on the duration of the experiment. In addition, 0.6 mg/ml BrdU (B5002-5G, Sigma-Aldrich) was administered in the drinking water. For DNA vaccination, mice were injected intradermally on each flank with 12.5 µg (total 25 µg) of vaccine encoding plasmids in saline solution, and electroporated over the injection site. Both inguinal LNs were harvested for analysis of DNA immunized mice.

### Flow cytometry

A recombinant HA (PR8) protein with a Tyr^98^-Phe^98^ substitution,^24^ and with a carboxy terminal 6x histidine tag was affinity purified on an anti-HA (H36-4-52, kind gift from Siegfried Weiss)^50^ chromatography column. GB113, a clonotype-specific mAb recognizing the pId^315^:I-E^d^-specific TCR of TCR-Tg mice,^51^ was affinity purified in the laboratory, conjugated to phycoerythrin, and used at 5 µg/ml. A TCR mimetic (TCRm), a scFv molecule recognizing the pId^315^:I-E^d^ complex, was produced, affinity purified, biotinylated, and used at 10 µg/ml. The following reagents were used from BD Biosciences (Franklin Lakes, NJ, USA): CD45.2 (560695) 7 µg/ml (561874) 10 µg/ml, B220 (552771) 7 µg/ml, IgD (565348) 7 µg/ml, CD138 (553714) 5 µg/ml, CD95 (740367) 7 µg/ml, CD154 (740685) 7 µg/ml, CXCR5 (560528) 7 µg/ml, MHCII (553623) 5 µg/ml, TCR Vβ 8 (553861) 5 µg/ml, CD14 (553739) 5 µg/ml, and BrdU staining kit (552598); eBioscience (San Diego, CA, USA): CXCR4 (17-9991-82) 7 µg/ml, CD69 (25-0691-82) 7 µg/ml, and CD49b (11-5971) 5 µg/ml; Tonbo biosciences (San Diego, CA, USA): CD4 (75-0041) 5 µg/ml, Ly-6G (35-1276-U100) 5 µg/ml, CD19 (35-0193) 5 µg/ml, CD3 (75-0032) 5 µg/ml, and CD11b (65-0112) 7 µg/ml; BioLegend (San Diego, CA, USA): CD64 (139306) 4 µg/ml, CD11c (117318) 4 µg/ml, F4/80 (123130) 5 µg/ml, GL7 (144603) 4 µg/ml (144614) 5 µg/ml, CD38 (102718) 4 µg/ml, PD-1 (135224) 7 µg/ml, MHCII (107622) 7 µg/ml, CD69 (104522) 7 µg/ml, CD19 (115520) 5 µg/ml, and streptavidin–phycoerythrin (405204) 4 µg/ml; Thermo Fisher Scientific: CD49b (15-5971-82) 5 µg/ml, PNA (L21409) 10 µg/ml, and Cell Trace CFSE (C34554) 5 µM; Abcam (Cambridge, UK): Anti-6x His tag antibody (ab133714) 6 µg/ml; Southern Biotech (Birmingham, AL): CD86 (1735-09) 5 µg/ml. All experimental setups included fluorescence minus one (FMO) stains, where the specific mAb was substituted with an isotype and fluorochrome-matched control. All samples were analyzed using an Attune NxT flow cytometer (Thermo Fisher Scientific) and FlowJo software (BD Biosciences).

### Calcium flux assay

Calcium flux assay was performed using Fluo-4 Direct™ Calcium Assay Kit (F10471, Thermo Fisher Scientific). Negatively enriched anti-Id B cells (2.5 million/ml) were loaded with Fluo-4 Direct™ calcium reagent according to the manufacturer’s instructions. Cells were incubated at 37 °C for 5 min before recording baseline for 30 s, then 500 nM ligand was added and the sample was recorded for 300 s (around 1.25 million cells). Samples were acquired on BD LSR II flow cytometer (BD Biosciences).

### Phosphotyrosine western blot and blot analysis

Negatively enriched anti-Id B cells were stimulated at 37 °C with 500 nM different vaccine proteins for different periods of time. For each time point, around 4 million cells were sampled. Cells were lysed for 45 min at 4 °C in NP-40 lysis buffer mixed with freshly prepared 0.5 mM phenylmethylsulfonyl fluoride (P-470, GoldBio, St. Loius, MO, USA), 1 mM sodium orthovanadate (450243, Sigma-Aldrich), and protease inhibitor cocktail (P8340, Sigma-Aldrich). Denatured samples were run on a 4–12% gel (NW04125BOX, Novex, Carlsbad, CA, USA) and transferred to a PVDF membrane (IB24001, Invitrogen). The membrane was blocked with 3% BSA (805095, Bio-Rad, Hercules, CA, USA) or milk powder (A0830, AppliChem, Darmstadt, Germany) in PBS-T, and incubated with either anti-phosphotyrosine (clone 4G10) (1.0 µg/ml) in 3% BSA or anti-actin (612656, BD Biosciences) (0.5 µg/ml) in 3% milk powder. Primary antibodies were detected with goat anti-mouse IgG-HRP (1030-05, Southern Biotech) and developed with ChemiGlow West Chemiluminescence Substrate Kit (60-12596-00, Proteinsimple, San Jose, CA, USA). Bands were quantified by gel analysis plugin in ImageJ version 2.0.0. The lanes were defined with a rectangular selection tool, and blot lane profile plots generated. The quantified area under the curve for all bands was normalized to the quantified area under the curve for the actin signal (loading control). All uncropped gels and blots are in Supplementary Fig. 8.

### Immunofluorescence staining

Spleens were embedded in OCT mounting medium (00411243, Q Path, VWR, Radnor, PA, USA) and frozen on dry ice. Tissues were kept at −80 °C. Five-micrometer sections were collected on glass slides and air dried. Sections were fixed in room temperature acetone for 5 min, air dried, and blocked in 30% normal rat serum with FcRγ blocking reagent (10 µg/ml, HB-197). Sections were then incubated with TCR clonotype-specific mAb GB113-Phycoerythrin (1.6 µg/ml) and anti-GL7-FITC (144604, BioLegend) (2.5 µg/ml) in blocking buffer. Signal was amplified using anti-FITC-Alexa Fluor 488 (A-11090, Thermo Fisher Scientific) (1.0 µg/ml) and anti-R Phycoerythrin-Texas Red (ab34734, Abcam) (1.0 µg/ml) diluted in PBS with 1% BSA. Sections were mounted with ProLong Diamond Antifade Mountant (P36970, Thermo Fisher Scientific). The number of GCs, counted as GL7^+^ cells with adjacent Id-specific T cells (GB113^+^), was quantified by counting 120 10x frames in the microscope for each group, ensuring that the same area of spleen had been investigated for each group.

### Enzyme-linked immunosorbent assays

Blood was harvested by puncture of the saphenous vein, and sera prepared by two successive centrifugations at 17.000 × g for 5 min at room temperature. ELISA plates (Costar 3590, Corning, NY, USA) were coated overnight at 4 °C with 1.0 µg/ml M315, blocked with 1% BSA, and incubated with serially diluted serum samples (*n* = 3–4 mice /group, as indicated in figure legends) or with supernatants from in vitro proliferation assays. Anti-Id antibodies were detected using either anti-mouse IgG1-bio (553500, BD Pharmingen, San Diego, CA, USA) 2 µg/ml, anti-mouse IgG2a-bio (553502, BD Pharmingen) 2 µg/ml, anti-mouse IgG2b-bio (553393, BD Pharmingen) 2 µg/ml, or anti-mouse IgG3-bio (HB128, ATCC) 2 µg/ml, followed by incubation with streptavidin-alkaline phosphatase (RPN1234, GE Healthcare, Buckinghamshire, UK) 1:1000, or alkaline phosphatase-conjugated goat anti-mouse IgG (A1418, Sigma-Aldrich) 1:5000 or anti-mouse IgM (A9688, Sigma-Aldrich) 1:2000. Plates were developed with phosphatase substrate (P4744, Sigma-Aldrich). Anti-Id IgG1, IgG2a, IgG2b and IgM standards for measurement were affinity purified in the laboratory. HA serum and avidity ELISAs were coated with 0.5 µg/ml recombinant HA [A/Puerto Rico/8/34 (H1N1)] (11684-V08H, Sino Biological, North Wales, PA, USA) and serum antibodies were detected with alkaline phosphatase conjugated goat anti-mouse IgG (A2429, Sigma-Aldrich) 1:5000. In the avidity ELISA, plates were washed with 6 M urea (U5128, Sigma-Aldrich) for 10 min before detection. CXCL13 ELISA was performed with the CXCL13/BLC/BCA-1 Quantikine ELISA Kit (MCX130, Abingdon, UK) according to kit instructions.

### ELISPOT assays

LNs (bilateral inguinal) and bone marrow cells from both tibia were harvested from vaccinated BALB/c mice (*n* = 4 per group) and single cell suspensions were prepared. The cells were used in an IL-4 ELISPOT assay (Mouse IL-4 ELISpot^PLUS^ (3311-4APW, Mabtech, Nacka Strand, Sweden), performed according to kit instructions. Cells were stimulated with HA(PR8) MHC class I (IYSTVASSL) or class II (SVSSFERFEIFPK or HNTNGVTAACSHEG)-restricted peptides (Proimmune, Oxford, England), or full-length HA [A/Puerto Rico/8/34 (H1N1)] (11684-V08H, Sino Biological). For the B-cell ELISPOT, MultiScreen HTS filter plates (MSIPS45, Merck Millipore Ltd., Tullagreen, Ireland) were coated with 0.5 µg/well of recombinant HA (PR8) (11684-V08H, Sino Biological) overnight at 4 ℃. Bone marrow suspensions were seeded and incubated for 20 h. Spots were detected with anti-mouse IgG (A1418, Sigma-Aldrich) 1:5000 and developed with phosphatase substrate (P4744, Sigma-Aldrich). ELISPOT plates were analyzed in CTL-ImmunoSpot® analyzer (CTL, Shaker Heights, OH, USA).

### Curve fitting and statistical analysis

Experimental values from the ELISAs were calculated by interpolating from the linear part of the standard curves. Antigen-binding dilution curves obtained in flow cytometry were fitted with a one-site total binding least squares fit. Background was constrained to the average value detected against the irrelevant antigen OVA. Statistical significance of fitted values was calculated by extra sum-of squares test. In vitro proliferation and serum dose–response experiments were investigated for statistical significance with an unpaired two-tailed student’s *t* test. Statistical analysis of flow cytometry data, ELISAs, and ELISPOTS were performed using a two-tailed Mann–Whitney test. All analysis was performed using GraphPad Prism v.6 software.

### Reporting summary

Further information on experimental design is available in the Nature Research Reporting Summary linked to this article.

## Supplementary information

Supplementary Information

Reporting Summary

## Data Availability

The data that support the findings of this study are available from the corresponding author upon reasonable request.

## Supplementary information

**Supplementary information** accompanies the paper on the *npj Vaccines* website (10.1038/s41541-019-0101-0).
