# Supplementary material for: Enhanced germinal center reaction by targeting vaccine antigen to major histocompatibility complex class II molecules
Source: NPJ Vaccines. 2019 Feb 11;4:9. doi: 10.1038/s41541-019-0101-0 (PMC6370881; doi:10.1038/s41541-019-0101-0)
Supplement: Supplementary file 1 — Supplementary Information [file 41541_2019_101_MOESM1_ESM.pdf]

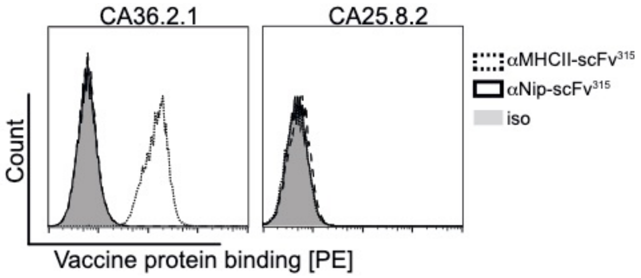

Supplementary figure 1. Binding of vaccine protein to MHCII. Binding of vaccine proteins to L-cell transfectants expressing relevant [CA36.2.1 ($E_{\beta}^{d}E_{\alpha}^{k}$)}, or irrelevant [CA25.8.2 (D^{d})] MHCII haplotypes measured in flow cytometry.

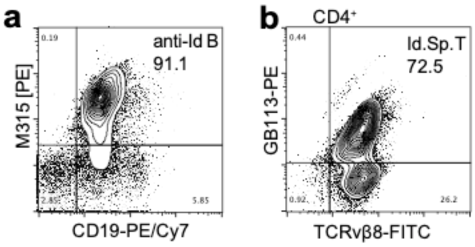

Supplementary figure 2. Antigen specific T and B cells. Negatively enriched anti-Id B cells from anti-Id^{DKI} mice and Id-specific T cells from TCR transgenic mice. (a) Anti-Id BCR binds the Id^{+} M315 [1]. (b) Transgenic TCR ($\alpha_T\beta_T$) is detected by the GB113 clonotype specific mAb [2]. About 25% of T cells in TCR transgenic mice express an endogenous TCR α chain (α_E). $\alpha_E\beta_T$ and $\alpha_T\beta_T$ TCR CD4^{+} cells are detected by anti-TCRvβ8 mAb.

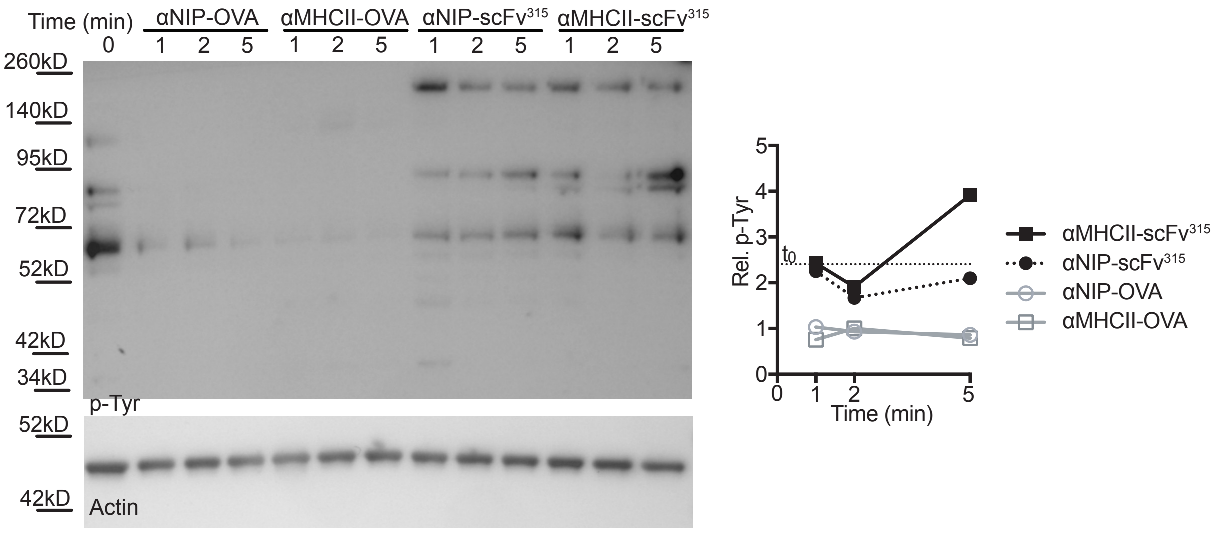

Supplementary figure 3. Phosphorylation after B cell ligation with vaccine proteins. Negatively enriched anti-Id B cells were incubated with the indicated vaccine proteins for the indicated periods of time. Cells were lysed and the amounts of phosphotyrosine measured in a Western Blot. The intensity of the bands was quantified from the blot. Relative phosphotyrosine represents the area under the peaks normalized to loading control, analyzed in ImageJ. The blots are from the same experiment and are processed in parallel.

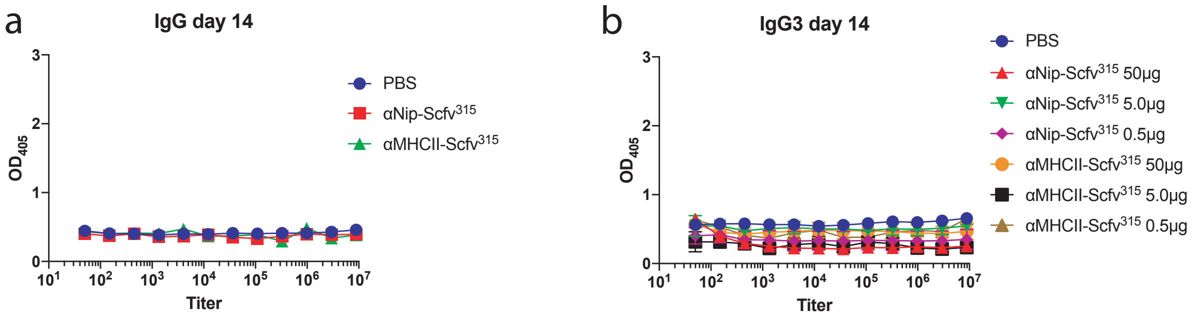

Supplementary figure 4. Anti-Id IgG levels in serum at day 14 in groups receiving different amounts of vaccine proteins. BALB/c mice received 4x10^5 anti-Id B and Id-sp T cells i.v. and were vaccinated with indicated proteins 24h later. (a) Levels of anti-Id IgG after transfer of 0.05µg of vaccine protein. (b) IgG3 levels in serum after transfer of indicated doses of vaccine proteins. Data points and error bars are mean±SEM, n=4mice/group.

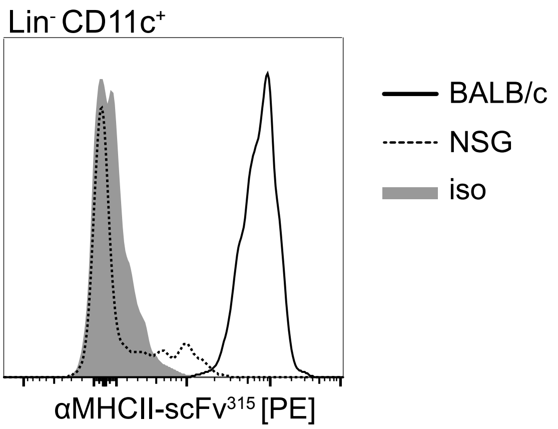

Supplementary figure 5. Vaccine protein binding to DCs from NSG and BALB/c mice. Splenocytes from NSG (I-A^{g7}) or BALB/c (I-A^{d} and I-E^{d}) mice were stained with vaccine proteins and binding to DCs (Lin^{-} $CD11c^{hi}$) were characterized in flow.

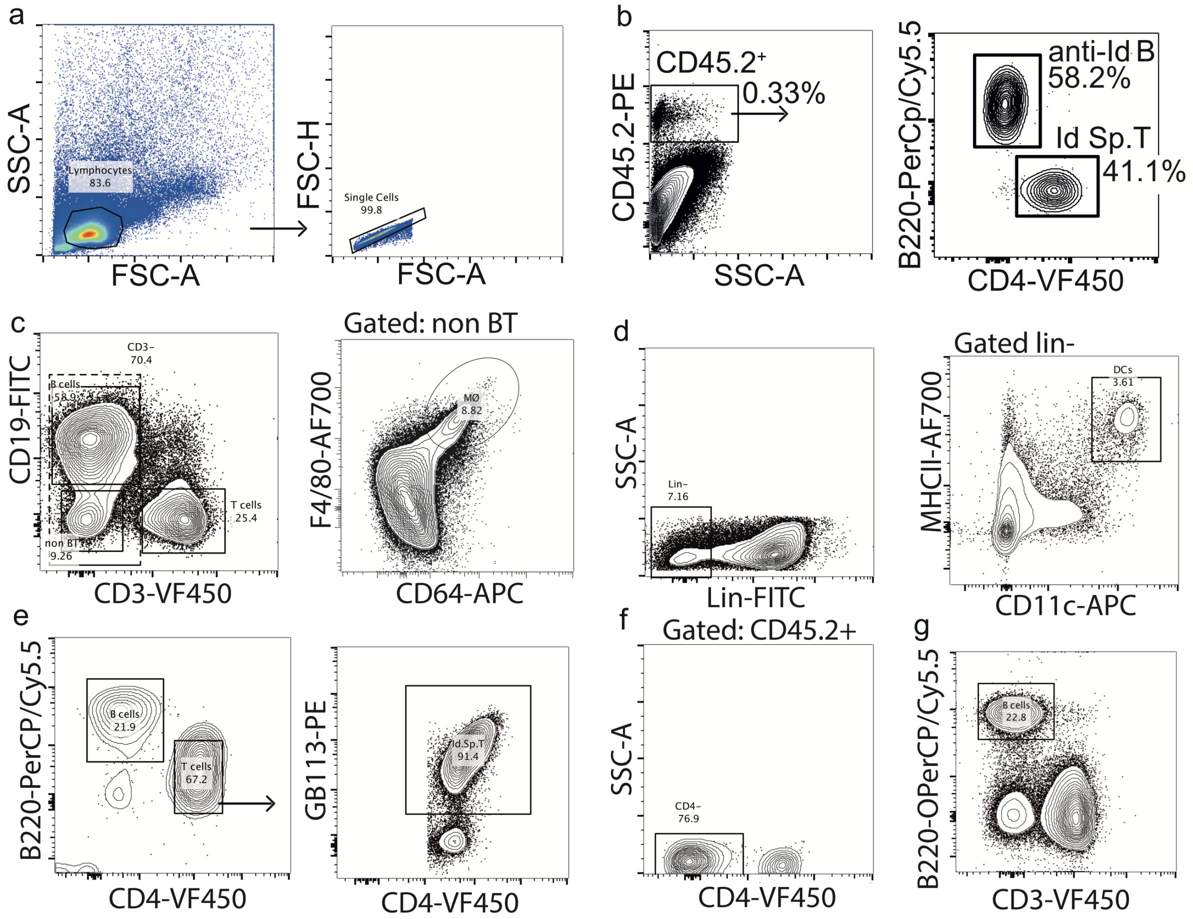

Supplementary figure 6. Exemplified gating strategy. (a) All flow cytometry samples were gated on size and single cell scatter as exemplified. (b) Exemplified gating strategy and

identification of Id-specific T cells and anti-Id B cells transferred to CD45.1 congenic hosts, identified as CD45.2^{+} CD4^{+}, and CD45.2^{+} B220^{+}, respectively. This gating was used in figure 5 and figure 6 b), d), and e). (c) Gating strategy for identification of B cells, T cells, non B or T cells, CD3^{-} cells (dashed line), and macrophages. This gating was used in figure 1 e), figure 2 e), and f). (d) Gating strategy for identification of DCs. This gating was used in figure 1 e), figure 2 f). (e) Gating strategy to identify B and T cells and CD4^{+} GB113^{+} T cells used in figure 3 i). (f) Gating strategy for identification of CD45.2^{+} CD3^{-} cells used in figure 6 c). (g) Gating strategy for identification of B220^{+} cells used in figure 7 b).

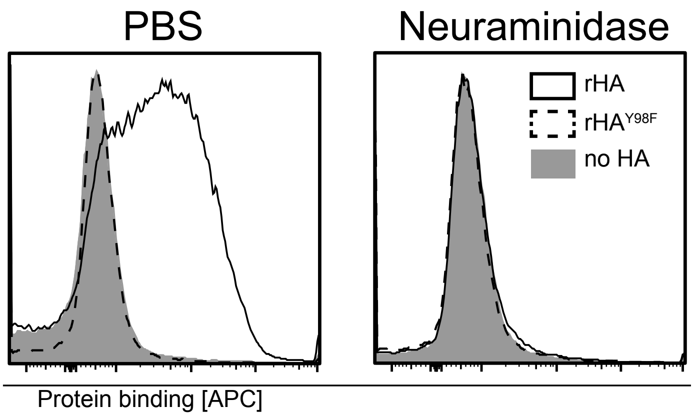

Supplementary figure 7. Abolished binding to sialic acid receptors in HA probe. MDCK cells were treated with neuraminidase or PBS for 1h at 37℃ and stained with recombinant HA (PR8). rHA^{Y98F} has a Tyr^{98}-Phe^{98} substitution, involved in the receptor binding site to sialic acid [3]. Binding of the HA probe to MDCK cells was detected with anti-6xhistidine-APC mAb.

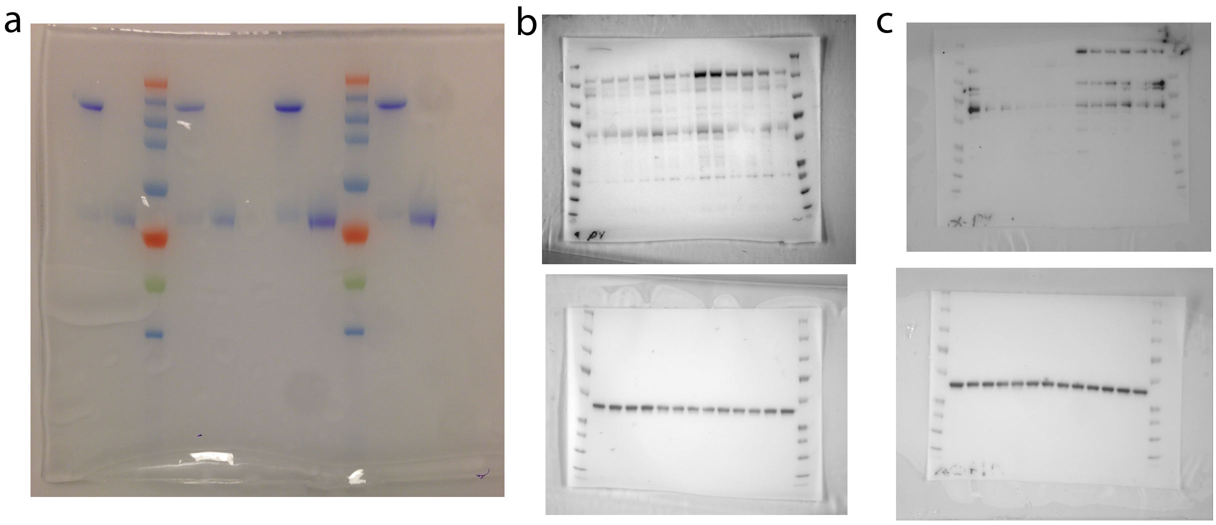

Supplementary figure 8. Uncropped gels and blots. Left lanes in the gel in (a) is shown in figure 1b. Blots in (b) are shown in figure 2c. Top blot is stained with anti p-Tyr and lower blot is stained with anti-actin (loading control). The blots are derived from the same experiment and were processed in parallel. Blots in (c) are shown in supplementary figure 3. Top blot is stained with anti p-Tyr and lower blot is stained with anti-actin (loading control). The blots are derived from the same experiment and were processed in parallel.

1. Jacobsen, J. *et al.* Naive idiotope-specific B and T cells collaborate efficiently in the absence of dendritic cells. *J. Immunol.* **192**, 4174-4183 (2014).

2. Bogen, B., Gleditsch, L., Weiss, S. & Dembic, Z. Weak positive selection of transgenic T cell receptor-bearing thymocytes: importance of major histocompatibility complex class II, T cell receptor and CD4 surface molecule densities. *Eur. J. Immunol.* **22**, 703-709 (1992).

3. Whittle, J.R. *et al.* Flow cytometry reveals that H5N1 vaccination elicits cross-reactive stem-directed antibodies from multiple Ig heavy-chain lineages. *J. Virol.* **88**, 4047-4057 (2014).
